# Significance of peak height velocity as a predictive factor for curve progression in patients with idiopathic scoliosis

**DOI:** 10.1186/1748-7161-10-S1-O16

**Published:** 2015-01-19

**Authors:** Masaaki Chazono, Takaaki Tanaka, Katsuki Kono, Nobumasa Suzuki, Keishi Marumo

**Affiliations:** 1 Orthopaedic Surgery Utsunomiya National Hospital, Japan; 2 Department of Orthopaedic Surgery, The Jikei University School of Medicine, Japan; 3 Department of Orthopaedic Surgery, Eiju General Hospital, Japan; 4 Scoliosis Center, Medical Scanning Tokyo, Japan

## 

Much attension has been paid to peak height velocity (PHV) as a possible predictor of curve progression in patients with idiopathic scoliosis (IS). Recently, we have developed the mobile application software HV scoliosis, which is downloadable by Google play or App store, to quickly calculate height velocity values without a complex formula in the clinical setting. The aim of this study was to analyze the relationship between the magnitude of the Cobb angle at PHV and the most recent treatment method before maturity in female patients with IS.

A retrospective review identified 56 skeletally immature female IS patients with a mean age of 10 years. These patients were followed until maturity and assigned to 1 of 3 groups: observation (O-group), brace treatment (B-group), and surgery (S-group), depending on the treatment method in use at the final follow-up visit. Height measurements were recorded at each visit; height velocity was calculated as the height change, in cm, divided by the time interval, in years. The PHV, age at PHV (APHV), height at PHV (HPHV), and final height (FH) were determined for each group. The sensitivity, specificity, and area under the curve (AUC) of the receiver-operating -characteristic (ROC) analysis were calculated to predict spinal curve progression for various Cobb-angle cutoff values at PHV.

The PHV had a mean value of 7.84, 8.61, and 8.94 cm/year in the O-group, the B-group, and S-group, respectively. The APHV was 11.7, 12, and 11 years, the HPHV was 153.2, 152.8, and 149.3 cm, and the FH was 161.5, 159.5, and 159.3 cm, respectively. When a Cobb angle of 32 degrees was used as the cutoff for determining which patients underwent surgery, ROC analysis revealed 78% sensitivity, 82% specificity, and an AUC of 0.925, acceptable values for curve progression in patients with IS.

These findings indicate that 32 degrees of spinal curvature when patients are at PHV is a significant predictive indicator for progression of the curve to a magnitude requiring surgery. We suggest that the curve-progression risk assessment in patients with IS should include PHV, along with measures of skeletal and non-skeletal maturities.

